# Rapid Solid-Liquid Dynamic Extraction (RSLDE): A Powerful and Greener Alternative to the Latest Solid-Liquid Extraction Techniques

**DOI:** 10.3390/foods8070245

**Published:** 2019-07-05

**Authors:** Daniele Naviglio, Pierpaolo Scarano, Martina Ciaravolo, Monica Gallo

**Affiliations:** 1 Department of Chemical Sciences, University of Naples Federico II, via Cintia; Monte S. Angelo Complex, 80126 Naples, Italy; 2 Department of Science and Technology, University of Sannio, Via Port’Arsa 11, 82100 Benevento, Italy; 3 Department of Molecular Medicine and Medical Biotechnology, University of Naples Federico II, via Pansini 5, 80131 Naples, Italy

**Keywords:** solid-liquid extraction, green extraction, RSLDE, bioactive compounds, Naviglio extractor, Naviglio’s principle

## Abstract

Traditionally, solid-liquid extractions are performed using organic and/or inorganic liquids and their mixtures as extractant solvents in contact with an insoluble solid matrix (e.g., the Soxhlet method) or using sequential atmospheric pressure systems that require long procedures, such as maceration or percolation. The objective of this procedure is the extraction of any compounds that can be carried out from the inner solid material to the outlet, resulting in a solution containing colorants, bioactive compounds, odorous substances, etc. Over the years, in the extraction techniques sector, there have been many important changes from the points of view of production, quality, and human and environmental safety due to improvements in technology. In more recent times, the interest of the scientific community has been aimed at the study of sustainable processes for the valorization of extracts from vegetables and food by-products, through the use of non-conventional (innovative) technologies that represent a valid alternative to conventional methods, generally through saving time and energy and the formation of fewer by-products. Therefore, with the development of principles based on the prevention of pollution, on a lower risk for human health, and on a low environmental impact, new systems have been implemented to reduce extraction times and solvent consumption, to improve efficiency, and to increase the productivity of the extracts. From this point of view, rapid solid-liquid dynamic extraction (RSLDE), performed using the Naviglio extractor, compared to traditional applications, is a technique that is able to reduce extraction times, generally leads to higher yields, does not require heating of the system, allows one to extract the active ingredients, and avoids their degradation. This technique is based on a new solid-liquid extraction principle named Naviglio’s principle. In this review, after reviewing the latest extraction techniques, an overview of RSLDE applications in various research and production sectors over the past two decades is provided.

## 1. Introduction

Solid-liquid extraction processes, both traditional ones (maceration and percolation) and those introduced more recently (e.g., supercritical fluid extraction (SFE) and accelerated solvent extraction (ASE), are based on two fundamental principles: diffusion and/or osmosis. On the basis of these principles, it is possible to make some general forecasts in relation to the extractive system, and it is possible to roughly hypothesize the extraction times and yields with respect to a generic solid matrix (generally vegetable) [1]. Three variables are to be optimized to achieve the best extractive conditions: by decreasing the “granulometry” of the solid, the extractive yield increases because of an increased surface area of contact between solid and liquid; the raising of the “temperature” of the system reduces the time of extraction due to the increase in diffusion phenomena (Fick’s law); the increase of the “affinity” of the extraction liquid towards the compounds to be extracted increases the effectiveness of the extraction process (*similis similia solvuntur*). However, the extractive principles which these techniques are based on have no active effect on the characteristics of the process, such as extraction times, yield, and efficiency. In fact, once the conditions have been set, the system reaches an equilibrium condition that can change only by modifying some parameters, such as the temperature or the addition of other extraction liquid [2]. For this reason, it is suggested that the extractive batch must be mixed during extraction to avoid a partial extraction due to the slow diffusion of compounds extracted.

Though solid-liquid extraction is a technique that has been known for a long time and is still widely used, there are still many unknown aspects that require further investigation to fully understand the mechanism. In the field of solid-liquid extraction techniques, it is possible to distinguish conventional extraction techniques, including maceration, percolation, squeezing, counter-current extraction, extraction through Soxhlet, and distillation, from unconventional (or innovative) ones. Conventional extractions have been used for many years, although they have many drawbacks: they require the use of high quantities of expensive and pure solvents, since during the process they consume a high amount; they have a low selectivity of extraction; they have a high solvent evaporation rate during the process; and they are generally characterized by long extraction times and by the thermal decomposition of thermolabile compounds [3,4]. To overcome all these limitations, new and promising solid-liquid extraction techniques, which are defined as non-conventional, have been introduced, mainly in the industrial field, such as ultrasound-assisted extraction (UAE) [5], supercritical fluid extraction (SFE) [6], microwave-assisted extraction (MAE) [7], extraction with accelerated solvent [8], solid phase microextraction [9], enzyme-assisted extraction [10], and rapid solid-liquid extraction dynamic (RSLDE) via the Naviglio extractor [11]. On the other hand, the interest of the scientific community has recently been aimed at the study of sustainable processes, so all these extraction techniques have common objectives, including the extraction of active ingredients (bioactive compounds) from the vegetable matrices, as well as their by-products for the valorization of waste, to improve the selectivity of the processes, to isolate the bioactive compounds in more suitable forms for detection and separation, and to provide an effective and reproducible method that is independent of the variability of the sample matrices; furthermore, high extraction yields are preferable to promote the economy of the process [12,13,14].

However, the extraction procedure generally takes place in a single solution (a single-step process), and it is difficult to set two or more extraction stages, because of the rise in extractant volume and time. Only the Soxhlet extractor limits the solvent volume, because it uses the distillation of the solvent, and the process works with fresh solvent. This can be considered a multi-step extractive process. Vice versa, RSLDE is based on a different principle. In fact, “the generation, with a suitable solvent, of a negative pressure gradient between the outside and the inside of a solid matrix containing extractable material, followed by a sudden restoration of the initial equilibrium conditions, induces forced extraction of the compounds not chemically linked to the main structure of which the solid is made” [11]. RSLDE changes the philosophy of solid-liquid extraction; the extraction happens thanks to a negative gradient of pressure between the inner material and the outside of the solid matrix (high pressure inside and low pressure outside; Naviglio’s principle). When the gradient of pressure is removed, the liquid flows out of the solid in a very fast manner and carries out all substances not chemically bonded to the main structure of the solid. This means that in this case the extraction is an “active” process because the gradient of pressure forces out the molecules, while techniques based on diffusion and osmosis are “passive” processes because the molecules are not forced out of the matrix.

According to this principle, the solid-liquid extraction process is first of all independent of the affinity that the compounds to be extracted from the solid matrix have towards the extracting solvent: they are, in fact, extracted by a difference of pressure between the liquid inside the matrix and the liquid on the outside of it. They are extracted out of the solid with a suction effect and can therefore also be extracted in solvents with opposite or different polarity. Furthermore, the pressure effect on the solid matrix and following the de-pressure leads to an active action with respect to the extraction process, as a small quantity of material is extracted at each pressure and depression cycle (the “active” solid-liquid extractive process), the extent of which is closely correlated with the pressure difference generated between the inside and the outside of the solid matrix and to the features of the solid matrix. Based on this new and innovative extractive principle, it has been made possible, in many cases, to use water as an extraction solvent, a condition that cannot be achieved with traditional techniques, such as maceration and percolation; in this case, the fermentative process is slower due to the movement of liquid around the solid, and this prevents the microorganisms from growing [15].

This review aims to give a brief overview of the various extraction techniques, focusing mainly on RSLDE and its various fields of application thanks to the introduction of an innovative solid-liquid principle of extraction.

## 2. State of the Art of Solid-Liquid Extraction Techniques

Solid-liquid extraction techniques are the basis of many analytical procedures for the preparation of samples and are reported in the official methods of analysis [16]. On the other hand, they are applied to the production of small quantities of homemade extracts such as alcoholic beverages and herbal teas [17,18]. These extraction procedures are also applied to industrial production. In fact, in many industrial processes, the initial phase of the preparation of a product requires the application of a solid-liquid extraction technique to isolate the extractable material contained in the most varied solid matrices, mainly vegetables. An important example is represented by medicinal plants, from which active ingredients with pharmacological properties are obtained; related fields are those of herbal medicine, cosmetics, and perfumery, which are the most ancient applications. In other industrial sectors such as the beverage industry, a solid-liquid extraction is used to obtain alcoholic extracts of fruit peels, flowers, leaves, etc., which are then mixed with water and sugar to obtain the finished product. The list could continue by referring to multiple industrial applications that are very similar.

The solid-liquid extraction is based on a simple phenomenon: if a solid matrix containing extractable compounds is immersed in a liquid, the latter begins to enrich itself with certain chemically related substances that move from the inside to the surface of the solid and then from the surface into the liquid. This principle is based on diffusion and osmosis and is performed by maceration, which is the simplest and most economical extraction technique and is therefore widely used. The maceration process requires only a closable glass or stainless steel container in which extractable solid is covered with liquid. To overcome the rapid saturation of liquid strictly around the solid, desultory agitation is required. Unfortunately, it is not always applicable, because it requires long contact times between the solid and the liquid; for example, plants cannot be macerated in water at room temperature for a very long time due to rotting phenomena. The production needs of the industry, which require the obtaining of large volumes of extracts in a short amount of time, have found an application in percolation extraction; in this case it is possible to process large quantities of solid material with large volumes of liquid and obtain the extract quite quickly, albeit sacrificing the efficiency of the extraction, which remains low due to the limited contact between the solid and the extracting liquid [19]. In this case, the solid matrix is not completely exhausted and could be re-extracted with another technique.

For special applications, such as the production of essential oils and, in general, compounds with low vapor pressure, it is possible to resort to steam distillation [20]. This solid-liquid extraction technique is particular in that it requires the transport of volatile compounds through a steam flow; since the isolated product is an essential oil, it can be considered a solid-liquid extraction technique. In any case, the extraction system is subjected to strong heating; therefore, the thermolabile compounds undergo transformations and consequently are not kept intact. As a result of this, steam distillation is not often applicable.

These examples serve to indicate that each of the solid-liquid extraction techniques that are currently used are not universally applicable since they are limited. Moreover, the extractive principle on which they are based is essentially linked to the phenomena of diffusion and osmosis of the substances contained in the solid, which tend to occupy the entire volume of the extracting liquid, after extraction. Therefore, desultory agitation of the extraction batch is necessary. To increase the efficiency of these extraction systems and to reduce the time of extraction, a temperature increase is used, which affects the increase in diffusion (Fick’s law), in order to reduce extraction times and increase yields. Generally, this expedient is not often applicable (over 40 °C) to vegetable matrices, because they contain substances that degrade due to heat, especially active principles [21,22].

The use of ultrasounds for the extraction of active ingredients from medicinal plants leads to the same results as extraction by pressing (squeezing). Furthermore, the system heats up due to the prolonged treatment, the solid matrix is completely crushed, and a mixture that is very difficult to separate into its constituents is obtained. Among other things, the use of ultrasound energy of more than 20 kHz may have an effect on the active phytochemicals through the formation of free radicals [23]. However, due to its speed, its economic advantage, and the relatively low-cost technology involved, UAE is one of the techniques used in the industry for bioactive compound extractions. As a result, in many cases, ultrasounds can be a good alternative to pressing because it simplifies the extractive system [24].

An alternative extraction technique is based on the use of supercritical fluids, mainly based on the use of carbon dioxide. In the supercritical phase, carbon dioxide assumes the characteristics of a non-polar solvent and is comparable to liquid n-hexane; with this method, it is therefore possible to extract non-polar compounds from solid matrices. The advantage of this technique is that, at the end of the extraction, the solvent, the carbon dioxide, is removed in the form of gas, enabling the possibility of recovering the concentrated extracted compounds with a very low environmental impact (green extraction). This technique finds applications at an industrial level, such as the extraction of oil from seeds, caffeine from coffee, nicotine from tobacco, etc. [25], but it is still very expensive and not universally applicable due to the difficulty of changing the polarity of carbon dioxide and for the interference of water contained in solids [26].

Another extraction technique is Soxhlet extraction, which is reported as an official extraction method [27] for numerous analytical methods in which an initial preparation of a solid sample extract is expected. The Soxhlet method also uses system heating, since it is based on the principles of diffusion and osmosis, so it cannot be used for substances that degrade due to heat [28]. Soxhlet extraction is a good method for the extraction of high boiling substances such as polycyclic aromatic hydrocarbons (PAH), polychlorobiphenyls (PCBs), dioxine, triglycerides, and so on. Nowadays, an improved method to perform Soxhlet extraction is named Soxtec [29]; this process is based on the same principles; however, thanks to pressure control, it is possible to accelerate the recirculation of the extractant solvent. In this way, the process is about 10 times faster [30].

To increase extraction yields and reduce time, accelerated solvent extraction (ASE) can be used [31]. This technique is based on an increase in diffusion because it is possible to extract solids by using liquids operating above their boiling temperature while being maintained in a liquid state by the increase in pressure. The material to be extracted is placed in a cylindrical steel container, and the extracting solvent is introduced; the temperature of the system is raised above the boiling point of the solvent, which is maintained in the liquid state thanks to a simultaneous increase in pressure (the vial is sealed to resist high pressure values: 100–200 bar). After a short contact period, the solid matrix is completely extracted. With this technique, it is not possible to extract thermally unstable compounds [32].

**Figure 1:**
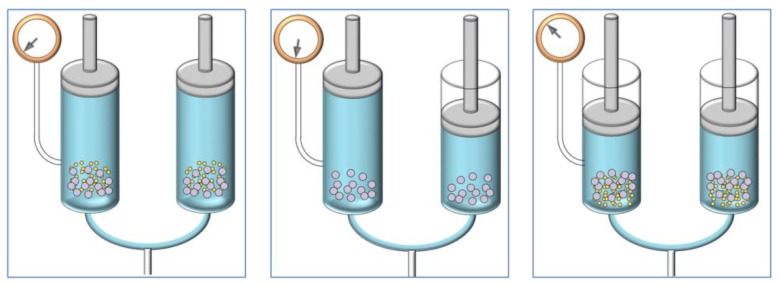
Schematic representation of the Naviglio extractor consisting of two extraction chambers connected via a conduit: the first two images show the dynamic phase, while the third image the static phase.

In this paper, a review of innovative solid-liquid extraction technology is presented, which can be used as a valid alternative to the existing ones, RSLDE, which can be considered a green means of extraction. The application of green technology aims to preserve the natural environment and its resources, and to limit the negative influence of human involvement [33]. The philosophy of green chemistry is to develop and encourage the utilization of procedures that reduce and/or eliminate the use or production of hazardous substances. The extraction takes place for the generation of a negative pressure gradient from the inside towards the outside of the solid matrix, so it can be carried out at room temperature, or even sub-environmental temperatures [11]. The functioning of this innovative system is based on a new solid-liquid extractive principle, as it is not equivalent to others reported in literature. The patent of the instrument named the Naviglio extractor was released in 2000 [34] and registered in 1998. An extractive cycle consists of both static and dynamic phases. During the static phase, the liquid is maintained under pressure at about 10 bar on the solid to be extracted and is left long enough to let the liquid penetrate inside the solid and to balance the pressure between the inside and the outside of the solid (about 1–3 min). After this, at the beginning of the dynamic phase, the pressure immediately drops to atmospheric pressure, causing a rapid flowing of liquid from the inside to the outlet of the solid matrix. At this moment, there is a suction effect of the liquid from the inside towards the outside of the solid. This rapid displacement of the extracting solvent transports the extractable material (compounds not chemically linked) outwards. The cycles can be repeated until the solid runs out. Experimental tests carried out to date on more than 200 vegetables have shown that, working at a pressure of about 10 bar, most solid matrices, regardless of the degree of crumbling, can be extracted using about 30 extractive cycles (two-minute static phase; two-minute dynamic phase) that are completed in two hours. Furthermore, the reproducibility of the extraction on the same matrix in terms of yield was proven, and experiments were carried out to compare this method with other extraction techniques, which showed that RSLDE had a higher recovery and a higher quality of extract, and in no case was the alteration of thermolabile substances induced [11] (Figure 1).

## 3. Comparison between the Various Solid-Liquid Extraction Techniques: Pros and Cons

The choice of methods and technologies related to an extraction process based on solid-liquid contact is not simple and this depends largely on the structural complexity and composition of the solid matrix; therefore it is not easy to find universal methods suitable for every type of Solid-Liquid extraction. In choosing the most appropriate techniques, operating conditions, solvents, etc., knowledge of the chemical properties of the compounds to be extracted and their behavior in the presence of different solvents is of fundamental importance. Due to the large extent of vegetables, operating conditions (granulometry of solid, different extractant liquids and their mix, temperature etc.) to the date, numerical and/or mathematical models that could anticipate the time and yield of extraction starting from precise conditions (solid type, solvent, temperature and so on) are not available. Alongside the aforementioned classical techniques, over the years others have been added; more complex and efficient and based on innovative extraction principles, such as extraction with supercritical fluids (SFE), ultrasound extraction (UAE), microwave extraction (MAE), accelerated solvent extraction (ASE) and finally the rapid solid liquid dynamic extraction (RSLDE) that uses the Naviglio extractor, which due to its characteristics of efficiency and improvement compared to other extraction techniques, was the subject of this review. RSLDE is an interesting new and innovative Solid-Liquid technology because it changes the philosophy of extraction; diffusion and osmosis are negligible in respect to the extraction based on difference in pressure between the inner material and the outlet of the solid matrix; this makes the extractive process “active” because it forces molecules out of solid. Below are the positive and negative aspects, pros and cons, of the main Solid-Liquid extraction techniques, nowadays existing, and briefly summarized in Table 1.

### 3.1. Squeezing

Squeezing is an ancient extraction technique based on extraction from vegetables of substances such as dyes, perfumes, poisons, and even substances with marked healing properties [35]. The technique is very simple, as it consists of a pressing system based on the application of pressure, by pestles, mortars, mullers, presses, etc., on the mass of a plant material; this mechanical action serves to obtain the exudates of vegetables in which important substances are contained [36]. This technique is unusual because liquid is not used to extract molecules from the inner material of a solid, but in spite of this it is counted among Solid-Liquid extraction techniques for the final effect of obtaining extracts. Squeezing finds its greatest use in the food industry, particularly in the extraction of oil from seeds and oleaginous fruits (olive oil, sunflower oil, etc.) and for the extraction of essential oils from the fruits of the genus *Citrus*. The advantage of squeezing is that it does not use any thermal gradient, which can induce the peroxidation of the extracted oils. Crushing solids releases elements contained in a vegetable contaminated by a series of undesired compounds. The product resulting from this process is rarely used as it is; in most cases, it is necessary to resort to a sophisticated separation processes in order to isolate the desired compounds. For this reason, despite being an ancient technique, applications are limited in number. Because the application of squeezing to other vegetables does not produce useful extracts, the need to discover new technology became a priority. In a paper by Vongsak et al. (2013), varying extraction methods, such as squeezing, decoction, maceration, percolation, and Soxhlet extraction have been used to extract phenolics and flavonoids from fresh and dried leaves of *M. oleifera*. The results show that maceration was more advantageous than other methods for the extraction of phenolics and flavonoids with the highest antioxidant activity [37].

Another particular and specific Solid-Liquid extraction technique is enfleurage, which uses extractant liquid that is not in contact with any solid. It is based on the dissolution of aromatic compounds in a liquid located above a vegetable. In this way, volatile compounds that fill a closed environment solubilize in the liquid (generally n-hexane). This extraction process has been widely used in the cosmetic industry in ancient times. This process was based on the observation that some fats had the peculiarity of absorbing odors from delicate parts of vegetables, generally flowers, which were then used as perfumes. With enfleurage, excellent quality oils are obtained; however, being an extremely expensive method, it is used today for demonstration purposes only [38]. Compared to squeezing, enfleurage is an unusual Solid-Liquid extraction technique because there is no contact between components. Briefly, squeezing and enfleurage are two simple and ancient techniques of Solid-Liquid extraction that have few and limited applications; both techniques do not use Solid-Liquid contact and are not often applicable.

### 3.2. Maceration

Another simple and economic separation technique is maceration, which is carried out in steel containers that can have both small and large capacities (starting from a few liters to industrial-level amounts) and inert material both towards the solid matrix and the extracting solvent. This Solid-Liquid extraction technique is the first and the oldest that is based on diffusion and osmosis and, for this reason, is counted as the reference technique for many applications involving the extraction of active principles from officinal plants [39]. The solid to be extracted is introduced into the inert container and completely covered by the solvent. In order to obtain the most complete extraction possible, the container must be hermetically closed, and agitation of the batch is required in order to enable the diffusion of compounds extracted in the liquid and thus to avoid the equilibrium of extracted substances. The extraction process is generally quite long and requires days or even weeks to complete. In this extraction process, both diffusion and osmosis phenomena, strongly dependent on temperature, occur. The extraction process is sped up with the increase in temperature (Fick’s law) or, more recently, with the use of ultrasounds or microwaves that increase the kinetic energy of the molecules that are found within the solid matrix and potentially extractables. This technique is recommended for extracting soluble or thermolabile active ingredients and for those matrices that, when hot, can lose substances of therapeutic interest (active principles). Maceration requires only occasional agitation for the diffusion of substances that are extracted in the mass of the extracting liquid. It is useful to underline that it is necessary to carry out maceration with limited quantities of solvent in several cycles and to subject the extracted material to squeezing, so as to avoid a strong loss of active ingredients. In fact, a dried and ground vegetable matrix absorbs a certain amount of solvent and, depending on its absorbent capacity, retains a more or less high portion in which the active ingredients are dissolved. A study by Ćujić et al. (2016) indicated that maceration was an effective and simple technique for the extraction of bioactive compounds from chokeberry fruit, even if it requires a long extraction time [40]. The maceration technique, with regard to extraction in aqueous phase, presents some variations, as it is not possible to use water at room temperature to extract vegetables because microbiological processes of fermentation take place more rapidly than extractive processes. To remedy this inconvenience, the infusion can be used, which can be represented as maceration for very short periods of time (1–2 min or until it has cooled), and is obtained by immersing medicinal plants or solid foods in water boiling to extract the active ingredients. In this case, the extraction is certainly faster, but the degradation of thermally labile substances also becomes faster. Briefly, maceration is a good technique of Solid-Liquid extraction and is applied in many cases for the production of extracts from officinal plants [39]. It is easy to apply, but extraction times are too long. The loss of liquid in the solid matrix is also relevant.

### 3.3. Decoction

Another variation of the classic maceration is decoction, which is carried out by contacting the matrix with the solvent operating at boiling temperature, for a variable time up to 30 min. At the end of extraction, the liquid is filtered, and the squeezed liquid of the extracted matrix impregnated with the solvent is added to it. This technique is therefore reserved for compact materials that have thermoresistant active principles, in accordance with which extraction requires the intervention of heat. The metabolic and antioxidant profiles of 10 herbal preparations have been assessed, and data have shown that the infusion procedure positively affected the extractability of the phenolic compounds compared to decoctions [41]. However, decoctions, like infusions, are easily altered and have limited validity; in fact, their shelf life is very short, and for this reason the extracts (generally named tisanes) must be consumed immediately after production. The last variant of classical maceration is digestion, which consists of heating the matrix in contact with the solvent from 35 to 60 °C. This technique is used when moderate heat is allowed in order to increase the extraction power of the solvent: if the solvent used is very volatile, it is necessary that the container in which the digestion is carried out is provided with a suitable refluxing refrigerant system for the recovery and recycling of the solvent itself. Digestion yields are higher than those obtained in maceration even if, with cooling and resting, cloudiness and the formation of precipitates occur [42]. Briefly, the decoction is a valid method to extract the active ingredients or aromas from the parts of medicinal plants or foods that are harder, such as roots, seeds, bark or wood, but it is not suitable for thermolabile compounds.

### 3.4. Percolation

#### 3.4.1. Simple Percolation 

In simple percolation, a particular container (cylindric percolator) filled with the matrix is used. In this matrix, the extracting liquid is recirculated by the means of a pump. Percolators can be made of glass, enameled iron, porcelain, or steel, and the shape depends on the nature of the matrix to be extracted. The percolation involves a series of fundamental operations: A good grinding is required; in fact, the degree of pulverization greatly influences the efficiency and the extraction time. Preliminary humidification is necessary, as the particles of the matrix to be extracted in contact with the solvent tend to swell. In the absence of this operation, the interstitial spaces diminish, and the regular outflow of the liquid is thus prevented. It is necessary to fill the percolator after a layer of cotton and sand is placed on the bottom, with the aim to block the solid matrix and as a filtration element. The matrix is added in a uniform and compact manner, minimizing and humidifying the contents of the chromatographic column. No spaces are left in the solid matrix. Preventive maceration occurs, and this works to soften the tissues and facilitates extraction. Finally, the extracting liquid is added to the percolator head and comes into contact with the solid matrix, and this liquid exerts a dynamic solubilization action on the matrix. In this technique, diffusion and osmosis occur as they do in maceration; the difference is in the continuous movement of extractant liquid through the solid, and this constitutes the driving force of percolation. The leachate thus forms, after filtration on cotton or sand, comes out from the percolator and is collected.

#### 3.4.2. Continuous Percolation 

In this extraction technique, a series of percolators are used, and in these the matrix to be extracted is placed and continuously fed in a counter-current by leachates that are less rich in extracted substances and that come from successive percolators, where the matrix is in a more exhausted state. Passing from one diffuser to the other, the solvent will be increasingly enriched by the extractable components while the matrix becomes increasingly impoverished in solutes. In this way, a concentration gradient is guaranteed in each diffuser. By optimizing the soaking and distribution ratios, the percolation operation can take place using a series of a few diffusers (5–10). One work by Chanda et al. (2012) reports a comparison between three different methods for extracting antioxidants from *Syzygium cumini* L. leaves: sequential cold percolation, decoction, and maceration. The results show that the sequential cold percolation extraction method is the best method of extracting leaf antioxidants from this plant [43]. In general, percolation does not require trained personnel to perform extraction operations. Furthermore, temperature and/or ultrasounds and microwaves can accelerate the extraction process; however, as for maceration, it is necessary to make the same considerations regarding thermolabile substances. Moreover, it is worth noting that, if the Solid-Liquid contact is very fast, the yield of extraction is not high, and generally about 50% (*w*/*w*) of extractable substances are lost in the matrix by the end of the process. Briefly, percolation is a very fast way to extract active principles from vegetables, but the process is not exhaustive.

### 3.5. Soxhlet Extraction

The Soxhlet method was introduced to determine whether it was possible to extract with the same Solid-Liquid ratio without using great quantities of extractant liquid and using “fresh” liquid [28]. Through the Soxhlet apparatus, this was possible because the liquid in contact with the solid is consistently “fresh” because of its distillation from the boiling liquid in the flask.

The Soxhlet method is used to extract compounds with high thermic stability due to a high temperature (the boiling point of the solvent). These substances are concentrated during the extractive process. The main advantage of this device is the use of a minimum quantity of solvent, thanks to its continuous purification and distillation after each passage through the matrix. The material to be extracted is placed in a porous thimble placed in the extraction chamber, which is placed on a distillation flask in which the solvent to be heated is placed. As the liquid boils, its vapors rise along a side tube up to the refrigerant mounted on the extractor. The liquid obtained from the condensation of the vapors falls into the extraction chamber passing through the material contained in the porous thimble, filling it until it reaches the elbow of the lateral siphon. At this point, due to its weight, the percolated liquid is sucked into the underlying flask, from which it is distilled again. The cycle described above is repeated several times until the extraction is considered complete: in this way, it is possible to extract all the soluble material from the matrix always using the same volume of solvent previously loaded in the boiler, renewed continuously by the distillation process. A review by De Castro and Priego-Capote (2010) describes the advantages and shortcomings of this centenary technique as well as the attempts to improve its performance and the achievements reached. In addition, currently, automation of Soxhlet procedures opened the door to the commercialization of a number of different approaches [44]. However, the Soxhlet apparatus and similar equipment cannot be used for the extraction of matrices that contain thermolabile active principles; moreover, this apparatus is not scalable to an industrial dimension. Briefly, Soxhlet extraction is a good tool for the extraction of many classes of compounds at a laboratory level; in fact, it is reported in official methods of analysis for the extraction of fats from foods, for the extraction of IPAs and PCBs from soil, etc. The limit of this type of extraction is related to the high temperature of the solvent, which does not allow for the extraction of thermo-sensible substances. 

### 3.6. Steam Current Distillation (SCD)

SCD is a preferred method applied to the extraction of essential oils from vegetables. The solid matrix is placed in a distillation flask in which steam is forced to pass through; volatile compounds are moved in a condenser where they pass from gas to liquid form, and at the bottom of the condenser a container is placed. The principle of this technique is based on the fact that the vapor pressure of volatile substances allows them to be removed from the vegetable. For this reason, this technique of Solid-Liquid extraction is different because volatile compounds are not extracted in a liquid. However, like maceration and enfleurage, this technique is considered Solid-Liquid extraction for the final effect of extraction. 

Distillation can be simple (e.g., the traditional distillation of wine waste) or in a steam current; in the latter case, the process becomes faster and the yield is higher in comparison with simple distillation. The auxiliary fluid that is used to assist distillation is generally represented by water in the form of steam, as it is very simple to generate steam (steam generators), has a high latent heat value (for this reason this process is expensive), and it is also particularly suitable for extractions of essential oils from aromatic plants. In a paper by Wei et al. (2012), steam distillation extraction and one-step high-speed counter-current chromatography were applied to separate and purify some bioactive compounds from the essential oil of *Flaveria bidentis* (L.) Kuntze, and good yields and high purity of the compounds (96.8% on average) were obtained [45]. However, despite its many advantages, steam current distillation cannot be used for all classes of organic compounds since the temperatures reached in the treatment can still be critical for the integrity of some of the molecules involved. Briefly, steam current distillation is an ancient technique for the extraction of thermally stable volatile compounds contained in vegetables, particularly for the extraction of essential oils. It is the most common technique for the production of distillates and similar products worldwide.

### 3.7. Microwave-Assisted Extraction (MAE)

MAE is a fast and efficient extraction technique based on the use of microwaves to heat the sample/solvent mixture in order to facilitate and speed up the extraction of the analyte. It is essentially very similar to the maceration process, but the introduction of a source of microwaves contributes to accelerating the extractive process. Unlike traditional heat sources, which act on a surface, from which heat diffuses towards the inner layers of the matrix by conduction and convection, a microwave heat source acts on the entire volume (if the medium is homogeneous) or on localized heating centers, consisting of the polar molecules present in the product. Therefore, whereas with conventional heating some time is required to heat the container before the heat is transferred to the solution, the microwaves directly heat the solution and the solid matrix, and the temperature gradient is kept to a minimum. Currently, MAE is already widely used in the laboratory for the extraction of organic pollutants from different matrices and for the isolation of natural products. It allows for a considerable reduction in the process time and in the solvent volumes used with respect to the classical extraction conducted through Soxhlet extractors. Recently, the MAE of phenolics from pomegranate peels was studied by Kaderides et al. (2019) and the extraction efficiency was compared with that of ultrasound extraction. The obtained extracts presented a high antioxidant activity [46]. However, MAE has the disadvantages that the tested samples must be thermostable, and a filtration phase is necessary, which in some cases can be very complex. Briefly, the introduction of microwaves in the maceration batch is used to perform MAE. This process is more accelerated than maceration because microwaves immediately heat both the solid and the liquid. The increasing temperature accelerates the extractive process, but at the same time if the energy of microwave is too high it is possible to damage the solid matrix and transform the active principles [47].

### 3.8. Ultrasonic Assisted Extraction (UAE)

The ultrasonication technique consists of passing a series of ultrasound pulses with increasing intensity through titanium probes immersed in a liquid medium. The probes convert the pulsed electrical energy applied to their heads into a vibrational impulse, which in a gaseous medium is transformed into ultrasound, while in a liquid medium, due to its incompressibility, it becomes an implosion. The waves generated by the pressure impulse in these particular vibrational conditions can cause cavitation, a phenomenon that consists in the formation of millions of small bubbles, during the negative pressure phase, which can implode in one of the subsequent compression phases. The implosion of each bubble causes a sudden change in temperature and pressure within the latter. The collapse of the cavity near a liquid-solid interface, however, differs considerably from the cavitation in a homogeneous liquid. In fact, passing through a liquid, the ultrasound expansion cycles exert a negative pressure on the liquid, with the molecules moving away from each other: if the ultrasound is sufficiently intense, the negative pressure exceeds the tensile strength of the liquid molecules generating a cavity. Cavitation bubbles form in the pre-existing weak points of the liquid and inside the solid spaces. Both were filled with gas in the powdered matter and suspended. Micro-bubbles, prior to cavitation were suspended in the irradiated liquid. Thus, there were devastating effects on the cellular structure. At high intensities, then, due to an inertial effect, a small cavity can quickly develop during the expansion half-cycle and will not have time to re-compress during the compression half-cycle. The bubble thus formed in the following cycle will suffer the same effect and increase in size, and the phenomenon will be repeated in the subsequent cycles until the bubble reaches a critical size such that it collapses with an increase in thermal energy. Instead, at lower acoustic intensities, cavity development may occur with a slower process. Under these conditions, a cavity will oscillate in size until it reaches the critical dimension, defined as a resonant dimension, where it can efficiently absorb the energy coming from the ultrasonic irradiation. The frequency range of use of the ultrasound is outside the perception limit of the human ear. Sonication is a technique that is used in many fields: the most widespread laboratory applications are in the field of biomedical and pharmaceutical research to lyse bacteria or cells in culture, in the field of environmental analysis for the extraction of various molecules, in the cosmetic and pharmaceutical industry for the preparation of creams and emulsions, and in biotechnology for the homogenization of immiscible liquids and the solubilization of difficult compounds. In the extractive field, this technique uses ultrasound frequencies to break up the cellular structure and facilitate diffusion processes. Goula et al. (2017) have carried out comparative studies between ultrasound-assisted and conventional solvent extraction in terms of processing procedure and total carotenoid content extracted from pomegranate wastes. The efficiency of the technique made it possible to produce an oil enriched with antioxidants [48]. The use of ultrasound in solvent extraction is a good remedy for the inconveniences linked to diffusion, but it is not always efficient. Due to the high energy developed inside the extraction batch, the breaking of the cellular structure results in extracts very similar to that obtained with the squeezing technique; in fact, vegetables are finely dispersed in the extractant liquid, and the resulting mixture is complex, so filtration and separation of active principles are required. Moreover, extracted compounds suffer from the direct bombing of cavitation generated by ultrasounds and can undergo transformations resulting in the loss of their beneficial activity. Briefly, for UAE, there are many variables to consider in order to obtain a good yield, so often the development of the various parameters lengthens the experimentation time. In the extraction of active ingredients from plants, for example, the results are comparable to extraction by squeezing, if not worse, due to the heating of the system for a prolonged time. The solid matrix is completely crushed, and a mixture is impossible to separate in its constituents is obtained, which makes this technique difficult to apply on an industrial level. This technique is often used in a laboratory procedure for sample preparation [49].

### 3.9. Supercritical Fluid Extraction (SFE)

SFE is a recent and very complex Solid-Liquid extraction technology [50]. The technique is based on the possibility of being able to use an extraction solvent that is a fluid (usually carbon dioxide) with properties that are intermediate between those of gases and liquids, named a supercritical fluid. Through modest variations in temperature and/or pressure, it is possible to modulate the properties of gases in a wide range and use their criticality to control phase behavior in extraction/separation processes. In practice, above the critical temperature, it is possible to continuously regulate the solubility of the fluid over a wide range, either with a small change in isothermal pressure or with a small isobaric temperature change. This ability to regulate the solvent power of a supercritical fluid is the main feature on which the SFE systems are based. These solvents can be used to extract and then efficiently recover the selected products. Since supercritical fluids have density, viscosity, and other properties that are intermediate between those of the substance in the gaseous state and those of the substance in the liquid state, the first and obvious advantage of this technique is that at the end of the extraction process the carbon dioxide is brought to ambient temperature and pressure, and gasification consequently leaves the substances extracted from the solid matrix. This fact makes SFE a green technique for Solid-Liquid extraction. A second advantage is represented by the best transport speeds: although the densities of supercritical fluids approximate those of conventional liquids, their transport properties are closer to those of gases. For example, the viscosity is many orders of magnitude lower than that of liquids, and the same diffusion coefficients are 100 times larger than typical ones observed in conventional liquids. The choice of CO_2_ as a supercritical fluid offers the following advantages: it spreads through extractive matrices faster than typical solvents that have a larger molecular size; it is cheap and can be obtained easily; it has higher diffusion coefficients and lower viscosities than the liquid solvent; it has a strong permeability, so the extraction time can be considerably shorter than that required by extraction with a common solvent; it is odorless, non-toxic, does not burn, does not explode, and does not damage the ozone layer; the working temperature is close to room temperature (31.1 °C), particularly suitable for heat-sensitive material, which would be decomposed by heat treatment; recovery is simple and convenient and can be recycled without treatment; extraction and removal are combined in a single technique, significantly shortening processing times in a simple and convenient way; and it has a variable solvent power, depending on the selected operating conditions (pressure and temperature). The application of supercritical fluids in the extraction of bioactive compounds and their operative extraction conditions has been reported in a review by da Silva et al. (2016) [51]. Supercritical fluid technology offers features that overcome many limitations of conventional extraction methods. However, the limitation of this technique consists in the need for specialized equipment as well as a lower solubilizing capacity for water-soluble compounds, which can be solved in part by adding traces of polar liquids (methanol and acetone), and the request for specialized personnel for its use. Briefly, SFE is the best choice for the extraction of non-polar substances, such as nicotine from tobacco, caffeine from coffee, and oil from seeds, at an industrial scale; it is completely green extraction technology. Unfortunately, this technique is very expensive, requires specialized personnel, and is not versatile.

### 3.10. Accelerated Solvent Extraction (ASE)

ASE represents a useful and innovative approach for the extraction of a wide class of compounds from matrices of complex chemical-physical entity. The extraction of the analytes from the matrices takes place using a solvent kept in liquid phase at temperatures above the boiling temperature thanks to the application of high pressure. This means that this technique requires a stainless steel container that resists the high pressures generated by raising temperatures beyond the boiling point of the solvent. The increase in temperature, in fact, accelerates the desorption of the analytes (Fick’s law) from the sample and their solubilization in the solvent, allowing for an effective extraction in a short period of time. In the use of solvents under high temperature and pressure, it is possible to influence the extraction process by modifying some chemical-physical parameters of the solvent–matrix system. The greatest effect on extraction is given by the temperature, as it influences the physical properties of the solvent and the interaction between the liquid phase and the material raising the molecular diffusion. Less significant is the effect of pressure that, even at low values, facilitates the penetration of the solvent into the pores of the sample. The essential function for ASE is that of keeping the solvent in a liquid state during the process. An advantage in the use of liquids at high temperature under pressure, with respect to supercritical fluids, is the fact that the former has a greater solvent strength and that, being used in methods that involve extractions at atmospheric pressure, no modifications or preliminary tests are required for the evaluation of their extractive efficiency. The other advantage is represented by the fact that, using liquid solvents, there are no phase changes in the return of the system to atmospheric conditions and therefore there is no need for liquid or packed restrictors or traps for the recovery of the analytes from the extract [52]. In their study, Cai et al. (2016) investigated the extraction efficiency of anthocyanins from purple sweet potatoes using conventional extraction, UAE, and ASE. The results show that extraction efficiencies were opposite for anthocyanins, and phenolics/flavonoids for the three methods [8]. On the other hand, the limits of the ASE method are represented by a partial extraction because of the static system and a possible degradation of the active ingredients due to operative conditions. Briefly, ASE is a good technique for the extraction of thermally very stable substances because raising the temperature behind the boiling point of the solvent remained in contact with the solid and the liquid at high temperatures for the entire experimental period. Due to the high pressure generated in the system, this technique is only used for sample preparation at a laboratory scale (10–20 mL).

### 3.11. Rapid Solid-Liquid Extraction (RSLDE)

Introduced in 2000 [34], RSLDE, through the use of the Naviglio extractor, represents a valid alternative to all existing Solid-Liquid extraction techniques and brings significant advantages in obtaining high quality extracts. First of all, it is not necessary to heat the extractive system, as the action performed is mechanical. Current extraction techniques (percolation, Soxhlet, steam current distillation and ultrasound), based on the principles of osmosis and diffusion, require an increase in temperature to increase the extraction efficiency. In the case of thermolabile compounds, however, the increase in temperature contributes to their degradation, as reported below. RSLDE requires a few extraction cycles, about 30 (depending on the matrix, but always within hours), to bring a large number of vegetable matrices to complete exhaustion. Compared to maceration, the official extraction method for many processes, RSLDE has been proven to be both quick and comprehensive. Moreover, it is possible to use water as an extracting liquid for many applications thanks to the reduced extraction times, while the prolonged contact of the plant solid matrices with water is unthinkable for maceration. RSLDE is an inexpensive technique and requires minimal energy expenditure when compared with SFE or ASE, both of which, among other things, require the use of high temperatures. In a recent work by Posadino et al. (2018), RSLDE was used to obtain polyphenolic antioxidants from the Cagnulari grape marc. The results indicate Naviglio extraction, as a green technology process, can be used to exploit wine waste to obtain antioxidants that can be used to produce enriched foods and nutraceuticals high in antioxidants [53].

In summary, the main advantages of RSLDE are as follows: exhaustion in a short period of time, with solid matrices containing extractable substances, at low operating temperatures (environment or sub-environment); reproducibility of the extraction since there is a real possibility of standardizing extracts for their active ingredients, with a guarantee of the production of high-quality extracts. From a careful comparison between the main characteristics of each of the Solid-Liquid extraction techniques described above, it is possible to state that, at present, no technique simultaneously provides all of the advantages offered by RSLDE, in terms of granulometry of the solid material, of the solvent type, or of the yield, time, quality, and stability of the extract (Table 1). On the other hand, due to its ease of use, its low-energy consumption, and the speed of the extraction process, RSLDE can be used as an exploratory and research technique for solid matrices that are not yet known and can be used to deal with materials that must undergo processes of washing (this means that the substances extracted from the solid are not important and for this reason will be discharged), such as polymers with clathrates, cork, etc. RSLDE has been advantageously used in processes in which it is important to fix substances inside the solid, as in leather tanning operations where both chrome solutions and solutions containing natural tannins are used (data not yet published). Moreover, RSLDE finds important applications in the beverage industry for the preparation of many alcoholic drinks; extracts from the ethyl alcohol of citrus peels (lemon, mandarin, orange, etc.) and from tonics and bitters from herbal extracts have been derived. In the perfume industry, it is possible to obtain fragrant and aromatic plant extracts by replacing maceration with RSLDE; in the same way, formulations of preparations in cosmetics and in herbal medicine have been improved compared to classical techniques, and alterations in the extracts obtained can be determined and made less active. Briefly, the application of this technology is very simple. It requires little energy for functioning, the time of extraction is low (two hours is the reference time), and the active principles are not degraded. Moreover, it is possible to apply RSLDE at temperatures below room temperature.

**Figure 2:**
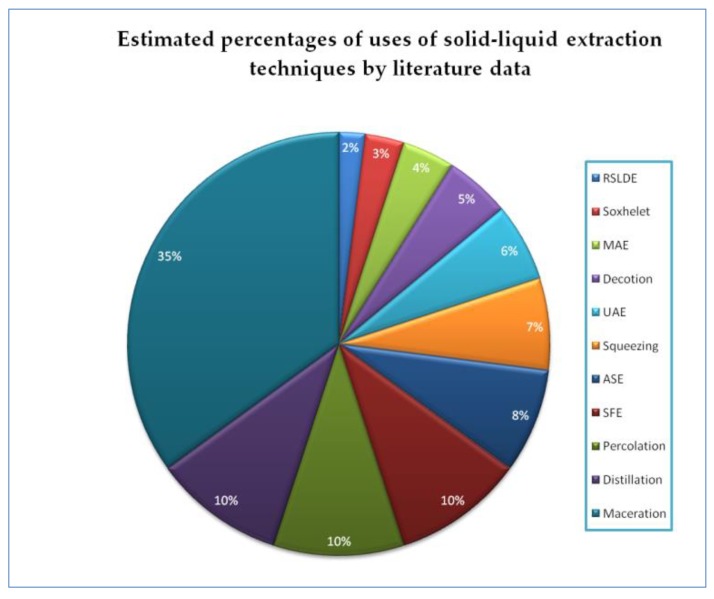
Pie chart showing the percentage of applications of each cited extraction technique.

Figure 2 shows a pie chart showing the application percentages of each technique mentioned in the work obtained from a qualitative analysis of the literature data.

## 4. RSLDE Applications in Various Industries 

### 4.1. The Pharmaceutical Sector

In the pharmaceutical sector, RSLDE has been used in the preparation of high-quality standardized extracts, including medicinal plant extracts and herbal extracts, fluid extracts, mother tinctures, glycerinated extracts, glyceric macerates, liposoluble extracts, bitter medicines, etc., all of which were obtained in a much shorter period of time (4–8 h) compared to maceration, which took 21 days (maceration data provided by the Official Pharmacopoeia) [39]. The speed of the process, the extraction at low temperature, and the high efficiency guarantee the total recovery of non-degraded active ingredients contained in medicinal plants [11].

*Paullinia cupana* seeds, commonly called guarana, are natural sources of phenolic antioxidants and antimicrobial compounds, and the use of guarana extract is interesting for the food, pharmaceutical, and cosmetic industries, where such natural additives are required [54]. A work by Basile et al. (2005) has reported extraction from *Paullinia cupana* var. *sorbilis* Mart. (*Sapindaceae*) seeds via RSLDE. Moreover, the antibacterial and antioxidant activity of the ethanol extract was assessed towards selected bacteria and in different antioxidant models [55].

*Cardiospermum halicacabum* is a herbaceous plant belonging to the *Sapindaceae* family, widely used in traditional medicine for its therapeutic properties. Menichini et al. (2014) analyzed the chemical composition of extracts from aerial parts and seeds, the inhibitory properties against some enzymes, and the antioxidant effects obtained using RSLDE and the Soxhlet method. The findings suggested the potential of both seeds and aerial parts of *C. halicacabum* for the treatment of neurological disorders [56]. Moreover, RSLDE was used to extract the flowering aerial parts of *Schizogyne sericea*, a halophytic shrub that is widespread on the coastal rocks of Tenerife (Canary Islands). The extracts obtained were assayed for in vitro biological activities. Results showed that aqueous extracts, rich in phenolic acids, were endowed with relevant radical scavenging activity [57].

Therefore, among the green extraction techniques used to improve the sensitivity and the selectivity of analytical methods, RSLDE represents a sustainable alternative to classical sample-preparation procedures used in the past [58]. In a study by Cozzolino et al. (2016), the extraction of curcuminoids by RSLDE was performed from *Curcuma longa* roots, focusing the interest on curcumin, the major phenolic component of the root that has been shown to have high antioxidant activity [59]. On the other hand, some studies have shown that curcumin exerts anti-tumor effects for its ability to induce apoptosis in cancer cells without cytotoxic effects on healthy cells. Moreover, some research has demonstrated an absence of toxicity in humans when dosing this active principle for short periods of time. Therefore, for its beneficial and healing properties, curcumin obtained by the described extraction method may be used as a natural dietary supplement [60]. A comparison between three extraction processes, including traditional maceration in n-hexane and ethyl alcohol, supercritical fluid extraction (SFE), and cyclically pressurized extraction (CPE), also known as RSLDE, has been carried out for the extraction of pyrethrins, predominantly nonpolar natural compounds with insecticidal properties found in pyrethrum, an extract of certain species of chrysanthemums [61].

### 4.2. The Cosmetic Sector

In cosmetics and perfumery, both in production and research, it is possible to produce extracts from vegetable matrices that contain pigments and odorous substances for the production and formulation of creams and perfumes. Officinal plants are raw, cosmetic materials that have been used in numerous formulations since ancient times. Plant extraction methods are carried out to obtain active phytocomplexes, both lipo and hydrosoluble [62]. The active ingredients of plants can be obtained from the plant complex or can be taken with drugs, a term that indicates the part or parts of the plant in which the active ingredients are present. Plant drugs are essentially whole plants (fragmented or cut), parts of plants, algae, fungi, or lichens in an untreated state, generally in dried form, but sometimes fresh. Phytocosmetic plants include vasal reinforcers (e.g., root ruscus, blueberry fruits, and gingko leaves), emollients (e.g., mallow, altea, and borage), stimulants (e.g., lavender, thyme, sage, juniper, and rosemary), bioactivators (e.g., calendula and carrot). They can be used as such or through their fluid extracts, and, with the addition of natural excipients, they can be used for natural functional cosmetics. The excipients are products that support and convey active and functional plant extracts. Essential oils or essences are an important part of phytocosmetics; they are obtained by the distillation of medicinal aromatic plants, obtaining a separation of the volatile component distillable from the non-volatile. These essential oils are diluted in appropriate solvents and applied in aesthetics according to their properties [63].

A review by Barbulova et al. (2015) reported some examples of the most important applications of agricultural food by-products in cosmetics and their performance as efficacy and safety [64]. In another review, Zappelli et al. (2016) showed examples of active cosmetic ingredients developed through biotechnological systems, whose activity on the skin has been scientifically proven through in vitro and clinical studies [65]. More recently, the reasons and the characteristics as well as the challenges of plant cell culture-based productions for the cosmetic and food industries are discussed in a review by Eibl et al. (2018) [66].

In the case of RSLDE, an active action is carried out towards the substances to be extracted; in fact, the compounds not chemically bound to the solid matrix are extracted in small quantities at each extraction cycle (active process) until the matrix is completely exhausted. The advantage is that the whole process takes place in the order of hours. The important consequences of the use of this technique are the possibility of extracting vegetable matrices with water. Therefore, it is possible to extract substances at temperatures even lower than room temperature for any thermolabile compounds. Moreover, these applications can be implemented on industrial, domestic, and lab scales [11].

### 4.3. The Herbal Sector

In the herbal and phytotherapy sector, both in production and research, RSLDE can be used for the extraction of plants and medicinal herbs for the production of fluid extracts. Since it is not necessary to heat the extraction system, it is possible to produce teas and/or infusions at room temperature, keeping the active ingredients unaltered.

Fresh plants of *Malva silvestris* were extracted with water using RSLDE, and the effects of terpenoids and phenol isolated from this plant on the germination and growth of dicotyledon *Lactuca sativa* L. (lettuce) were studied [67].

In a study by Ferrara et al. (2014), a conventional extraction technique (UAE) and a cyclically pressurized Solid-Liquid extraction (RSLDE) were compared, in order to obtain qualitative and quantitative data related to the bioactive compounds of saffron. The results obtained showed that extracts via RSLDE had significant advantages in terms of extraction efficiency and the quality of the extract [68].

### 4.4. The Food and Beverage Sector

In the food sector, both in production and research, RSLDE has been applied in various ways. Lycopene, the carotenoid responsible for the red color of many fruits and vegetables, is considered fundamental for its antioxidant action. Therefore, its extraction is of great interest in various sectors. In fact, it can be used both for the formulation of functional foods and in cosmetics. In addition, lycopene can be extracted from tomato processing waste using only water as an extract liquid. The use of water as an extracting phase considerably reduces the cost of the entire process when compared with the commonly used solvent-based procedure or with the newer supercritical extraction process of lycopene from tomato waste. Lycopene, not soluble in water, was recovered in a quasi-crystalline solid form and purified by solid-phase extraction using a small amount of organic solvent [69]. Lycopene can be used as a dye and/or a natural antioxidant. Moreover, through RSLDE, it is also possible to produce limoncello, a lemon liqueur, in just two hours, avoiding the long traditional maceration that takes 7–14 days [70]. Nowadays the industrial process for the production of lemon liquor is performed via maceration, as home-made products are made, and the process requires at least 48 h of lemon peel infusion in alcohol.

In a paper by Formato et al. (2013), two Solid-Liquid extraction techniques, supercritical fluid extraction (SFE) with and without modifiers and cyclically pressurized Solid-Liquid extraction or RSLDE, were compared on the basis of the extraction of acidic compounds contained in hops. The results showed that both techniques were valid for the extraction of α and β acids from hops. By suitably varying the parameters of the two extractive procedures, it was possible to obtain extracts for use in the production of beer and of dietary supplements and drugs [71].

In order to obtain the alcoholic extracts of some herbal mixtures, the traditional maceration procedure was compared to RSLDE. Three different mixtures of various parts of plants were extracted with both methods, and results were compared. Organoleptic tests performed on alcoholic bitters obtained from different extracts have been used to determine the optimum extraction time for the two different methods used. The results showed that the bitters produced with RLSDE were more appreciated than bitters prepared by maceration [72]. In another work by Naviglio et al., (2014), the extraction process for the production of *Cinchona calisaya* elixir starting from the same vegetable mixture was performed by a conventional maceration and RSLDE. The results show that, compared to the conventional method, RSLDE allowed for extraction at room temperature using cyclical extraction pressurization. In this way, it was possible to avoid thermal stress on thermolabile substances while simultaneously reducing extraction time [73].

Based on the Naviglio extractor, a system of extraction has been devised that can be used for student laboratory experiments to illustrate RSLDE (two-syringe system). In a paper by Naviglio et al. (2015), students compare two extraction techniques for the preparation of limoncello: maceration and the two-syringe system. The development of the two-syringe system for simple manual operations reduced the risks of the procedure, allowing students to evaluate the extraction efficiency of the two methods [74]. In another work, two extraction processes for the production of limoncello—the traditional maceration of lemon peels and RSLDE—were compared. Alcoholic extracts were analyzed by gas chromatography, and alcoholic extracts were analyzed by UV spectrophotometry to identify the more abundant chemical species, while the organoleptic tests performed on the final product (limoncello) provided an indication of the taste of the final product. Results showed that the RSLDE process was 120 times faster than maceration and had a greater efficiency in a short period of time [75].

The results of a recent study by Gigliarelli et al. (2017) indicated that RSLDE is an effective technique for the extraction of piperine from fruits of *Piper longum* compared to other techniques, such as Soxhlet extraction, decotion (International Organization for Standardization), and conventional maceration [76].

Stevioside and rebaudioside A are the main diterpene glycosides present in the leaves of the *Stevia rebaudiana* plant, which is used in the production of foods and low-calorie beverages. In this context, RSLDE constitutes a valid alternative method to conventional extraction by reducing the extraction time and the consumption of toxic solvents and favoring the use of extracted metabolites as food additives and/or nutraceuticals [77,78].

*Portulaca oleracea*, commonly known as purslane, is a wild plant pest of orchards and gardens, but is also an edible vegetable rich in beneficial nutrients. The purpose of a work by Gallo et al. (2017) was to compare various Solid-Liquid extraction techniques to determine the most efficient technique for the extraction of biomolecules from leaves of purslane. Therefore, extraction reproducibility was tested on the same matrix in terms of weighting, and comparison experiments were performed. RSLDE showed a higher recovery and a higher quality of extract, and in no cases did it induce the alteration of heat-sensitive substances [79].

RSLDE can be used for applications other than extraction, such as the quick hydration of legumes and the de-structuring of vegetables at room temperature (heat-free cooking) for a better preservation of nutritional elements. Hydrating beans before cooking reduces cooking time, increases their tenderness and weight, and improves their appearance after cooking. Naviglio et al. (2013) described a process of cyclically pressurized soaking for the rapid hydration of cannellini beans at room temperature. The hydration process was approximately 10-fold faster than the traditional soaking procedure, and the microbial load developed by the end of this process was much lower compared to that obtained using the traditional process [80].

A large amount of food waste and by-products is produced from farm to plate and represents valuable sources for the production of compounds with a high added value. Consequently, the application of innovative approaches is necessary due to the limitation of conventional processes. In this context, RSLDE can be a useful tool to increase extract yield and quality, reducing extraction time, temperature, and toxic solvents. In a study by Bilo et al. (2018), RSLDE was used for the synthesis of a new bioplastic produced from rice straw, an agricultural waste that is generally not recovered. The results show that, depending on the environmental humidity, the material shows a dual mechanical behavior that can be exploited to obtain shrink films and sheets or to drive a shape memory effect. Therefore, rice straw bioplastics could represent a new potential eco-material for different application fields [81]. In addition, RSLDE could provide an innovative approach to increase the production of specific compounds from food waste for use as nutraceuticals or as ingredients in the design of functional foods. As it is currently known, grape pomace is a by-product of winemaking that can be conveniently reused in many different ways, including agronomic use as well as cosmetic industry applications. Moreover, the by-products can also be used in the energy field as biomass for the production of biogas or for food plants used for the production of energy. As an added value, grape pomace resulting from the production of wine also contains numerous bioactive compounds. In a very recent work by Gallo et al. (2019), to extract polyphenols, grape peels were processed via RSLDE, which does not require the use of any organic solvent, nor does it include heating or cooling processes that can cause the loss of substances of interest [82]. Still, within the framework of the guiding principles for eco-innovation, which aims at a zero waste economy, many residues have the potential to be reused as raw material for new products and applications and in other production systems, such as those in the nutraceutical and pharmaceutical sectors. Another recent work by Gallo et al. (2019) shows an alternative process for the extraction of lycopene from tomato waste through RSLDE. The high purity of lycopene obtained using this procedure make the process very attractive, and the pure product obtained could be used in various applications [83].

## 5. Conclusions

The efficiencies of conventional and non-conventional extraction methods mostly depend on the critical input parameters, on understanding the nature of the matrices to be extracted, on the chemistry of the compounds, and on scientific expertise. In particular, RSLDE introduces a new Solid-Liquid extractive technology that is based on the difference in pressure between the inner material and the outlet of the solid matrix, which generates a suction effect (matter transfer) that causes the compounds that are not chemically linked to the solid matrix to be extracted. Therefore, RSLDE makes it possible to optimally replace most of the current Solid-Liquid extraction techniques and brings about considerable new features and advantages in obtaining high-quality extracts. It changed the concept of Solid-Liquid extraction based only on diffusion and osmosis phenomena (passive process of extraction) using a new philosophy that bases on the generation of pressure gradient between the inner material and the outlet of the solid (active process of extraction). In addition, the areas of application of RSLDE are numerous and include the pharmaceutical, cosmetic, herbal, and food and beverage industries. Furthermore, RSLDE can be used for the extraction of food waste, a by-product of various industrial, agricultural, domestic, and other food sectors, which is currently increasing due to the increase in these activities. These by-products can be used as a potential source of bioactive and nutraceutical compounds that have important applications in the treatment of various disorders.

## Figures and Tables

**Table 1:** Comparison and main characteristics of Solid-Liquid extraction techniques are herein presented.

Extraction Technique	Solvent	Granulometry	Time	Yield	Quality Extracted	Extract Stability	References
Squeezing	Indifferent	Not important	Minimum	Exhaustive	Poor	Poor	[35,36,37,38]
Maceration	Fundamental	Important	Long	Exhaustive	Great	Great	[39,40]
Decotion	Fundamental	Important	Long	Exhaustive	Great	Great	[41,42]
Percolation	Fundamental	Important	Middle	Partial	Good	Good	[43]
Soxhlet	Fundamental	Important	Long	Exhaustive	Poor	Poor	[28,44]
SCD	Indifferent	Not important	Middle	Partial	Poor	Poor	[45]
MAE	Fundamental	Not important	Middle	Partial	Poor	Poor	[46,47]
UAE	Fundamental	Not important	Middle	Partial	Great	Great	[48,49]
SFE	Indifferent	Not Important	Middle	Exhaustive	Poor	Poor	[50,51]
ASE	Fundamental	Not important	Minimum	Exhaustive	Poor	Poor	[8,52]
RSLDE	Indifferent	Not important	Minimum	Exhaustive	Great	Great	[53]

Abbreviations: SCD: steam current distillation; MAE: microwave-assisted extraction; UAE: ultrasound-assisted extraction; SFE: supercritical fluid extraction; ASE: accelerated Solid-Liquid extraction; RSLDE: rapid Solid-Liquid dynamic extraction.

## References

[1] Azmir J., Zaidul I.S.M., Rahman M.M., Sharif K.M., Mohamed A., Sahena F., Omar A.K.M. (2013). Techniques for extraction of bioactive compounds from plant materials: A review. J. Food Eng..

[2] Aguilera J.M. (2003). Solid-liquid extraction. Extraction Optimization in Food Engineering.

[3] Wang L., Weller C.L. (2006). Recent advances in extraction of nutraceuticals from plants. Trends Food Sci. Technol..

[4] Galanakis C.M. (2012). Recovery of high added-value components from food wastes: Conventional, emerging technologies and commercialized applications. Trends Food Sci. Technol..

[5] Chemat F., Rombaut N., Sicaire A.G., Meullemiestre A., Fabiano-Tixier A.S., Abert-Vian M. (2017). Ultrasound assisted extraction of food and natural products. Mechanisms, techniques, combinations, protocols and applications. A review. Ultrason. Sonochem..

[6] Khaw K.Y., Parat M.O., Shaw P.N., Falconer J.R. (2017). Solvent supercritical fluid technologies to extract bioactive compounds from natural sources: A review. Molecules.

[7] Ekezie F.G.C., Sun D.W., Cheng J.H. (2017). Acceleration of microwave-assisted extraction processes of food components by integrating technologies and applying emerging solvents: A review of latest developments. Trends Food Sci. Technol..

[8] Cai Z., Qu Z., Lan Y., Zhao S., Ma X., Wan Q., Li P. (2016). Conventional, ultrasound-assisted, and accelerated-solvent extractions of anthocyanins from purple sweet potatoes. Food Chem..

[9] Souza-Silva É.A., Jiang R., Rodríguez-Lafuente A., Gionfriddo E., Pawliszyn J. (2015). A critical review of the state of the art of solid-phase microextraction of complex matrices I. Environmental analysis. Trends Anal. Chem..

[10] Kumar S.J., Kumar G.V., Dash A., Scholz P., Banerjee R. (2017). Sustainable green solvents and techniques for lipid extraction from microalgae: A review. Algal Res..

[11] Naviglio D. (2003). Naviglio’s principle and presentation of an innovative solid–liquid extraction technology: Extractor Naviglio^®^. Anal. Lett..

[12] Barba F.J., Zhu Z., Koubaa M., Sant’Ana A.S., Orlien V. (2016). Green alternative methods for the extraction of antioxidant bioactive compounds from winery wastes and by-products: A review. Trends Food Sci. Technol..

[13] Chemat F., Rombaut N., Meullemiestre A., Turk M., Perino S., Fabiano-Tixier A.S., Abert-Vian M. (2017). Review of green food processing techniques. Preservation, transformation, and extraction. Innov. Food Sci. Emerg. Technol..

[14] Al Jitan S., Alkhoori S.A., Yousef L.F. (2018). Phenolic acids from plants: Extraction and application to human health. Studies in Natural Products Chemistry.

[15] Azwanida N.N. (2015). A review on the extraction methods use in medicinal plants, principle, strength and limitation. Med. Aromat. Plants.

[16] AOAC Method 43.290 (1990). Official Methods of Analysis of the AOAC.

[17] Willson K.C., Clifford M.N. (2012). Tea: Cultivation to Consumption.

[18] Liguori L., Russo P., Albanese D., Di Matteo M. (2018). Production of low-alcohol beverages: Current status and perspectives. Food Processing for Increased Quality and Consumption.

[19] Aspé E., Fernández K. (2011). The effect of different extraction techniques on extraction yield, total phenolic, and anti-radical capacity of extracts from *Pinus radiata* Bark. Ind. Crop. Prod..

[20] Božović M., Navarra A., Garzoli S., Pepi F., Ragno R. (2017). Essential oils extraction: A 24-hour steam distillation systematic methodology. Nat. Prod. Res..

[21] Joana Gil-Chávez G., Villa J.A., Fernando Ayala-Zavala J., Basilio Heredia J., Sepulveda D., Yahia E.M., González-Aguilar G.A. (2013). Technologies for extraction and production of bioactive compounds to be used as nutraceuticals and food ingredients: An overview. Compr. Rev. Food Sci. Food Saf..

[22] Rostagno M.A., Prado J.M. (2013). Natural Product Extraction: Principles and Applications.

[23] Esclapez M.D., García-Pérez J.V., Mulet A., Cárcel J.A. (2011). Ultrasound-assisted extraction of natural products. Food Eng. Rev..

[24] Gallo M., Ferrara L., Naviglio D. (2018). Application of ultrasound in food science and technology: A perspective. Foods.

[25] Jesus S.P., Meireles M.A.A. (2014). Supercritical fluid extraction: A global perspective of the fundamental concepts of this eco-friendly extraction technique. Alternative Solvents for Natural Products Extraction.

[26] Sánchez-Camargo A.D.P., Parada-Alonso F., Ibáñez E., Cifuentes A. (2019). Recent applications of on-line supercritical fluid extraction coupled to advanced analytical techniques for compounds extraction and identification. J. Sep. Sci..

[27] AOAC Method 963.15 (1990). Agricultural Chemicals, Contaminants, Drugs.

[28] Jensen W.B. (2007). The origin of the Soxhlet extractor. J. Chem. Educ..

[29] Anderson S. (2004). Soxtec: Its principles and applications. Oil Extraction and Analysis. Critical Issues and Competitive Studies.

[30] Carro N., Cobas J., García I., Ignacio M., Mouteira A., Silva B. (2018). Development of a method for the determination of SCCPs (short-chain chlorinated paraffins) in bivalve mollusk using Soxtec device followed by gas chromatography-triple quadrupole tandem mass spectrometry. J. Anal. Sci. Technol..

[31] Molino A., Rimauro J., Casella P., Cerbone A., Larocca V., Chianese S., Musmarra D. (2018). Extraction of astaxanthin from microalga *Haematococcus pluvialis* in red phase by using generally recognized as safe solvents and accelerated extraction. J. Biotechnol..

[32] He Q., Du B., Xu B. (2018). Extraction optimization of phenolics and antioxidants from black goji berry by accelerated solvent extractor using response surface methodology. Appl. Sci..

[33] Hilali S., Fabiano-Tixier A.S., Ruiz K., Hejjaj A., Nouh F.A., Idlimam A., Chemat F. (2019). Green extraction of essential oils, polyphenols and pectins from orange peel employing solar energy. Towards a Zero-Waste Biorefinery. ACS Sustain. Chem. Eng..

[34] Naviglio D. (2000). Rapid and Dynamic Solid–Liquid Extractor Working at High Pressures and Low Temperatures for Obtaining in Short Times Solutions Containing Substances that Initially Were in Solid Matrixes Insoluble in Extracting Liquid. Italian Patent.

[35] Baldwin E.A., Bai J., Plotto A., Cameron R., Luzio G., Narciso J., Ford B.L. (2012). Effect of extraction method on quality of orange juice: Hand-squeezed, commercial-fresh squeezed and processed. J. Sci. Food Agric..

[36] Armenta S., Garrigues S., de la Guardia M. (2015). The role of green extraction techniques in Green Analytical Chemistry. TrAC Trends Anal. Chem..

[37] Vongsak B., Sithisarn P., Mangmool S., Thongpraditchote S., Wongkrajang Y., Gritsanapan W. (2013). Maximizing total phenolics, total flavonoids contents and antioxidant activity of Moringa oleifera leaf extract by the appropriate extraction method. Ind. Crop. Prod..

[38] Stratakos A.C., Koidis A., Preedy V.R. (2016). Methods for extracting essential oils. Essential Oils in Food Preservation, Flavor and Safety.

[39] European Pharmacopoeia Commission (2014). European Pharmacopoeia.

[40] Ćujić N., Šavikin K., Janković T., Pljevljakušić D., Zdunić G., Ibrić S. (2016). Optimization of polyphenols extraction from dried chokeberry using maceration as traditional technique. Food Chem..

[41] Fotakis C., Tsigrimani D., Tsiaka T., Lantzouraki D.Z., Strati I.F., Makris C., Zoumpoulakis P. (2016). Metabolic and antioxidant profiles of herbal infusions and decoctions. Food Chem..

[42] Manousi N., Sarakatsianos I., Samanidou V. (2019). Extraction techniques of phenolic compounds and other bioactive compounds from medicinal and aromatic plants. Engineering Tools in the Beverage Industry.

[43] Chanda S.V., Kaneria M.J. (2012). Optimization of conditions for the extraction of antioxidants from leaves of *Syzygium cumini* L. using different solvents. Food Anal. Methods.

[44] De Castro M.L., Priego-Capote F. (2010). Soxhlet extraction: Past and present panacea. J. Chromatogr. A.

[45] Wei Y., Du J., Lu Y. (2012). Preparative separation of bioactive compounds from essential oil of *Flaveria bidentis* (L.) Kuntze using steam distillation extraction and one step high-speed counter-current chromatography. J. Sep. Sci..

[46] Kaderides K., Papaoikonomou L., Serafim M., Goula A.M. (2019). Microwave-assisted extraction of phenolics from pomegranate peels: Optimization, kinetics, and comparison with ultrasounds extraction. Chem. Eng. Process. Process Intensif..

[47] Chan C.H., Yusoff R., Ngoh G.C., Kung F.W.L. (2011). Microwave-assisted extractions of active ingredients from plants. J. Chromatogr. A.

[48] Goula A.M., Ververi M., Adamopoulou A., Kaderides K. (2017). Green ultrasound-assisted extraction of carotenoids from pomegranate wastes using vegetable oils. Ultrason. Sonochem..

[49] Tiwari B.K. (2015). Ultrasound: A clean, green extraction technology. Trends Anal. Chem..

[50] Sharif K.M., Rahman M.M., Azmir J., Mohamed A., Jahurul M.H.A., Sahena F., Zaidul I.S.M. (2014). Experimental design of supercritical fluid extraction–A review. J. Food Eng..

[51] Da Silva R.P., Rocha-Santos T.A., Duarte A.C. (2016). Supercritical fluid extraction of bioactive compounds. TrAC Trends Anal. Chem..

[52] Nayak B., Dahmoune F., Moussi K., Remini H., Dairi S., Aoun O., Khodir M. (2015). Comparison of microwave, ultrasound and accelerated-assisted solvent extraction for recovery of polyphenols from *Citrus sinensis* peels. Food Chem..

[53] Posadino A., Biosa G., Zayed H., Abou-Saleh H., Cossu A., Nasrallah G., Pintus G. (2018). Protective effect of cyclically pressurized solid–liquid extraction polyphenols from Cagnulari grape pomace on oxidative endothelial cell death. Molecules.

[54] Santana A.L., Macedo G.A. (2018). Health and technological aspects of methylxanthines and polyphenols from guarana: A review. J. Funct. Foods.

[55] Basile A., Ferrara L., Del Pezzo M., Mele G., Sorbo S., Bassi P., Montesano D. (2005). Antibacterial and antioxidant activities of ethanol extract from *Paullinia cupana* Mart. J. Ethnopharmacol..

[56] Menichini F., Losi L., Bonesi M., Pugliese A., Loizzo M.R., Tundis R. (2014). Chemical profiling and in vitro biological effects of *Cardiospermum halicacabum* L. (*Sapindaceae*) aerial parts and seeds for applications in neurodegenerative disorders. J. Enzym. Inhib. Med. Chem..

[57] Caprioli G., Iannarelli R., Sagratini G., Vittori S., Zorzetto C., Sánchez-Mateo C.C., Petrelli D. (2017). Phenolic acids, antioxidant and antiproliferative activities of Naviglio^®^ extracts from *Schizogyne sericea* (*Asteraceae*). Nat. Prod. Res..

[58] Bandar H., Hijazi A., Rammal H., Hachem A., Saad Z., Badran B. (2013). Techniques for the extraction of bioactive compounds from Lebanese *Urtica Dioica*. Am. J. Phytomed. Clin. Ther..

[59] Cozzolino I., Vitulano M., Conte E., D’Onofrio F., Aletta L., Ferrara L., Gallo M. (2016). Extraction and curcuminoids activity from the roots of *Curcuma longa* by RSLDE using the Naviglio extractor. ESJ.

[60] Pulido-Moran M., Moreno-Fernandez J., Ramirez-Tortosa C., Ramirez-Tortosa M. (2016). Curcumin and health. Molecules.

[61] Gallo M., Formato A., Ianniello D., Andolfi A., Conte E., Ciaravolo M., Naviglio D. (2017). Supercritical fluid extraction of pyrethrins from pyrethrum flowers (*Chrysanthemum cinerariifolium*) compared to traditional maceration and cyclic pressurization extraction. J. Supercrit. Fluids.

[62] Cabaleiro N., De La Calle I., Bendicho C., Lavilla I. (2013). Current trends in liquid–liquid and solid–liquid extraction for cosmetic analysis: A review. Anal. Methods.

[63] Ali B., Al-Wabel N.A., Shams S., Ahamad A., Khan S.A., Anwar F. (2015). Essential oils used in aromatherapy: A systemic review. Asian Pac. J. Trop. Biomed..

[64] Barbulova A., Colucci G., Apone F. (2015). New trends in cosmetics: By-products of plant origin and their potential use as cosmetic active ingredients. Cosmetics.

[65] Zappelli C., Barbulova A., Apone F., Colucci G. (2016). Effective active ingredients obtained through Biotechnology. Cosmetics.

[66] Eibl R., Meier P., Stutz I., Schildberger D., Hühn T., Eibl D. (2018). Plant cell culture technology in the cosmetics and food industries: Current state and future trends. Appl. Microbiol. Biotechnol..

[67] Cutillo F., D’Abrosca B., DellaGreca M., Fiorentino A., Zarrelli A. (2006). Terpenoids and phenol derivatives from *Malva silvestris*. Phytochemistry.

[68] Ferrara L., Naviglio D., Gallo M. (2014). Extraction of bioactive compounds of saffron (*Crocus sativus* L.) by Ultrasound Assisted Extraction (UAE) and by Rapid Solid-Liquid Dynamic Extraction (RSLDE). ESJ.

[69] Naviglio D., Pizzolongo F., Ferrara L., Naviglio B., Santini A. (2008). Extraction of pure lycopene from industrial tomato waste in water using the extractor Naviglio. Afr. J. Food Sci..

[70] Naviglio D., Pizzolongo F., Romano R., Ferrara L., Naviglio B., Santini A. (2007). An innovative solid-liquid extraction technology: Use of the Naviglio Extractor for the production of lemon liquor. Afr. J. Food Sci..

[71] Formato A., Gallo M., Ianniello D., Montesano D., Naviglio D. (2013). Supercritical fluid extraction of α-and β-acids from hops compared to cyclically pressurized solid–liquid extraction. J. Supercrit. Fluids.

[72] Naviglio D., Ferrara L., Formato A., Gallo M. (2014). Efficiency of conventional extraction technique compared to rapid solid-liquid dynamic extraction (RSLDE) in the preparation of bitter liquors and elixirs. IOSR J. Pharm..

[73] Naviglio D., Formato A., Gallo M. (2014). Comparison between 2 methods of solid–liquid extraction for the production of *Cinchona calisaya* elixir: An experimental kinetics and numerical modeling approach. J. Food Sci..

[74] Naviglio D., Montesano D., Gallo M. (2015). Laboratory production of lemon liqueur (Limoncello) by conventional maceration and a two-syringe system to illustrate rapid solid–liquid dynamic extraction. J. Chem. Educ..

[75] Naviglio D., Formato A., Vitulano M., Cozzolino I., Ferrara L., Zanoelo E.F., Gallo M. (2017). Comparison between the kinetics of conventional maceration and a cyclic pressurization extraction process for the production of lemon liqueur using a numerical model. J. Food Process Eng..

[76] Gigliarelli G., Pagiotti R., Persia D., Marcotullio M.C. (2017). Optimisation of a Naviglio-assisted extraction followed by determination of piperine content in *Piper longum* extracts. Nat. Prod. Res..

[77] Gallo M., Vitulano M., Andolfi A., DellaGreca M., Conte E., Ciaravolo M., Naviglio D. (2017). Rapid Solid-Liquid Dynamic Extraction (RSLDE): A new rapid and greener method for extracting two steviol glycosides (stevioside and rebaudioside A) from stevia leaves. Plant Foods Hum. Nutr..

[78] Gallo M., Formato A., Formato G., Naviglio D. (2018). Comparison between two solid-liquid extraction methods for the recovery of steviol glycosides from dried stevia leaves applying a numerical approach. Processes.

[79] Gallo M., Conte E., Naviglio D. (2017). Analysis and comparison of the antioxidant component of *Portulaca oleracea* leaves obtained by different solid-liquid extraction techniques. Antioxidants.

[80] Naviglio D., Formato A., Pucillo G.P., Gallo M. (2013). A cyclically pressurised soaking process for the hydration and aromatisation of cannellini beans. J. Food Eng..

[81] Bilo F., Pandini S., Sartore L., Depero L.E., Gargiulo G., Bonassi A., Bontempi E. (2018). A sustainable bioplastic obtained from rice straw. J. Clean. Prod..

[82] Gallo M., Formato A., Giacco R., Riccardi G., Lungo D., Formato G., Amoresano A., Naviglio D. (2019). Mathematical optimization of the green extraction of polyphenols from grape peels through a cyclic pressurization process. Heliyon.

[83] Gallo M., Formato A., Ciaravolo M., Langella C., Cataldo R., Naviglio D. (2019). A water extraction process for lycopene from tomato waste using a pressurized method: An application of a numerical simulation. Eur. Food Res. Technol..

